# Incorporating physical activity in the comprehensive care of people living with HIV starting antiretroviral therapy: Insights from a specialized care setting in São Paulo, Brazil

**DOI:** 10.1371/journal.pone.0254168

**Published:** 2021-07-01

**Authors:** Ardiles Vitor Santos, Elisabete Cristina Morandi Santos, Camila Melo Picone, Tulio Gamio Dias, Sandra Maria Lima Ribeiro, Alex Antonio Florindo, Aluisio Cotrim Segurado

**Affiliations:** 1 Department/Division of Infectious Diseases, Hospital das Clínicas, Faculdade de Medicina, Universidade de São Paulo, São Paulo, Brazil; 2 Prefeitura Municipal de Joinville, Santa Catarina, Brazil; 3 Escola de Ciências, Artes e Humanidades, Universidade de São Paulo, São Paulo, Brazil; 4 Faculdade de Saúde Pública, Universidade de São Paulo, São Paulo, Brazil; Pontificia Universidade Catolica do Rio Grande do Sul, BRAZIL

## Abstract

**Background:**

Managing HIV infection as a chronic condition includes encouraging adoption of healthy behaviors and promotion of physical activity (PA). However, people living with HIV (PLH) are often under social and programmatic vulnerability that may compromise behavior change. Understanding such barriers is crucial for successful incorporation of PA in their comprehensive care.

**Methods and findings:**

In this study, we describe PA, energy intake from diet, and anthropometry of a cohort of PLH starting antiretroviral therapy (ART) at a Brazilian reference clinic, report how PA was addressed in routine care and investigate association between PA, energy intake and psychosocial constructs that may facilitate PA (social support and self-efficacy for PA). Among 61 PLH (86.9% males, mean age = 32.5 years) anthropometry was normal, but 47.5% reported PA below recommendations. Despite presenting high social support scores, family encouragement for PA was low, and self-efficacy classified as medium. Chart reviews yielded infrequent reports concerning PA. After adjusting for gender and age, we found a negative association between energy intake from diet and self-efficacy, but none between PA and energy intake or between PA and psychosocial constructs.

**Conclusions:**

We conclude that patients in our cohort were insufficiently active when starting ART, and that PA was poorly addressed by caretakers in routine HIV care. Nevertheless, social support and self-efficacy scores suggest potential for behavioral change. Caregivers should therefore start considering patients’ vulnerabilities and establishing strategies to help them overcome barriers to incorporate PA in their comprehensive care effectively.

## Introduction

Combined antiretroviral therapy has remarkably reduced AIDS-related morbidity and mortality and enabled HIV infection to be managed as a chronic disease [1–3]. However, in this prolonged survival scenario people living with HIV (PLH) need to cope with long-term outcomes of chronic HIV infection, including lipodystrophy, diabetes and consequent increased cardiovascular risk [4].

Promotion of healthy lifestyles have thus become a priority for PLH under antiretroviral therapy (ART) as part of more comprehensive care approaches that include health promotion and engagement in physical activity (PA) [5, 6]. In fact, there is evidence that combined resistance and aerobic training improve body composition, increase muscular strength, and reduce blood cholesterol of PLH [7–9]. In addition, training leads to increased cardiorespiratory fitness and flexibility of these patients [10–13].

Brazil is known to have the largest number of PLH in Latin America and for playing a leading role in establishing public policies for HIV prevention and care within the national health system. Currently, ART is provided free of charge for over 700,000 PLH by the public health sector, following the “test-and-treat strategy”. Although recommendations for promotion of PA among PLH have been incorporated in national guidelines [14, 15], little is known about the challenges to engage faced by PLH starting ART in a megacity, such as São Paulo. Recent data show that the city is ranked last among Brazilian state capitals in general population engagement in PA, with only 34.6% of its residents reporting practicing exercise for more than 150 minutes weekly in their leisure time [16]. Given their particularly high social vulnerability, PLH are expected to have even more difficulties in adopting healthier lifestyles that include PA.

Our study aimed to provide evidence to help specialized HIV care centers recognize patients’ vulnerabilities and particular needs to overcome barriers and engage in PA as soon as they are admitted to services and start ART. Using several assessment tools, we describe current PA patterns, energy intake from diet and physical assessment in a cohort of PLH who start ART in a university HIV outpatient clinic in São Paulo. We also evaluate psychosocial constructs that have been recognized as capable of facilitating engagement in PA (social support and self-efficacy for PA) and explore how caregivers are addressing PA in routine patient care. Associations between current PA and other variables of interest (energy intake from diet, social support and self-efficacy for PA) were also sought after.

## Material and methods

### Study design and setting

A cross-sectional study was conducted at the HIV outpatient reference clinic affiliated to the Division of Infectious Diseases, Hospital das Clinicas, School of Medicine, University of São Paulo, where 3,200 PLH are currently under multidisciplinary clinical follow-up.

### Patient selection

From June 2014 to May 2016 and from December 2017 to March 2018 we recruited PLH aged 18 to 59 years old who had started ART at the clinic for no longer than 4 months. The first recruitment period coincided with patient selection for a randomized pragmatic trial that investigated the impact of a multicomponent PA program to prevent body changes and metabolic disturbances associated with ART [17], whereas the second period initiated after the trial was completed. We excluded pregnant women, men and women with waist circumferences exceeding 102 cm or 88 cm, respectively; patients with comorbidities (diabetes mellitus, uncontrolled hypertension, stroke or cancer) or metabolic abnormalities (fasting blood glucose concentrations >100 mg/dL, total cholesterol >239 mg/dL, LDL-cholesterol >160 mg/dL, triglyceride >150 mg/dL); patients using anabolic steroids, hypolipidemic or hypoglycemic medication; those who reported esthetic surgery in the prior 12 months and those with contraindications for PA, such as severe immunosuppression or musculoskeletal disorders.

### Data collection

We invited eligible subjects to take part in the study while waiting for a medical consultation at the clinic. After consenting, they were interviewed by a trained member of the research team in search of sociodemographic data and information about smoking and alcohol use. Additionally, the following data collection instruments were applied:

a) International Physical Activity Questionnaire–IPAQ–long version [18]: to assess current PA (performed in the previous week), including PA related to mobility; PA in leisure and sports; and physical exercises. Scores were calculated for each type of activity. A total score was obtained, adding the three partial scores and interpreted following the questionnaire guidelines. We then used metabolic equivalent data (MET) to calculate patient’s weekly energy expenditure in kilocalories.

b) Energy intake from diet: to quantify energy intake we used three 24h-food recalls, an instrument which is based on the patient’s description of his/her diet on the previous day, on a regular weekday and on a day during weekend or holiday [19]. Data analysis was performed using the Avanutri® Revolution software.

c) Social Support Scale for PA [20]: to assess support for PA under two hypothetical scenarios: mild PA (walking) and moderate to intense PA. For each scenario, patients were questioned about support provided by family members, or people living in the same household and by people living elsewhere. Data analysis was performed classifying participants as follows: scores <12 = low, 13–24 = medium, and 25–36 high social support for PA.

c) Self-efficacy scale for PA: to assess the confidence a patient has in performing PA or in changing his/her behavior regarding PA [21]. The tool evaluates how confident he/she is to engage in walking or in moderate to intense PA under five different scenarios: physical status, weather conditions, mood, and availability of time. A positive answer scores 1, whereas negative answers do not score. As such, maximum total 10 points can be obtained for each type of activity, yielding a total maximum score of 20. We adopted the following interpretation criteria: total score = 10: low, 11–15: medium, 16–20: high self-efficacy for PA.

d) Standardized physical evaluation: included assessment of heart rate (digital monitors), blood pressure (aneroid sphygmomanometer), body weight (digital scale) and height (stadiometer). Duplicate measurements were performed of arm, calf, waist, abdomen, and hip circumferences (anthropometric tape). We calculated the average of the duplicate measurements, waist-hip ratios, and BMIs, and interpreted them based on WHO standards [22, 23].

Finally, we reviewed patients’ charts in search of information concerning physical activity in medical histories or provider recommendations.

### Data analysis

For descriptive statistics of study variables, we used absolute numbers and frequencies for categorical data, and central trend and dispersion measures for quantitative variables. Associations between current PA (measured as weekly energy expenditure in kcal as the dependent variable) and energy intake from diet and psychosocial constructs (social support and self-efficacy) that facilitate PA were sought after using the maximum likelihood estimation (MLE) for robust linear regression analysis after adjusting for patients’ gender and age, using the *rlm* function (R software, version 3.5.1). A statistical significance of 0.05 was adopted throughout the analysis.

### Ethical issues

The study was approved by the Ethics Committee of the Faculty of Medicine, University of São Paulo (protocol approval–CAAE 74647217.0.000.0068). Subject participation was voluntary and followed informed consent. Patients’ anonymity and confidential data management were ensured throughout the study.

## Results

The study cohort comprised 61 PLH, most of them males who reported themselves as non-white. Their mean age was 32.5 years old (SD = 8.4). Detailed socio-demographic data are described in Table 1.

**Table 1:** Socio-demographic profile, smoking and alcohol use in the study cohort.

Variables	N	%
Gender		
Male	53	86.9
Female	8	13.1
Skin color		
White	27	44.3
Brown	26	42.6
Black	8	13.1
Site of birth		
São Paulo	30	49.2
Other states	31	50.8
Residence		
São Paulo city	44	72.1
Elsewhere	17	27.9
Occupation		
Worker	55	90.2
Student	1	1.6
On unpaid leave/unemployed	5	8.2
Marital status		
Single	42	69.0
Married or living with a partner	12	19.6
Divorced	7	11.4
Smoking		
Yes	20	32.8
No	41	67.2
Alcohol use		
1–2 times/week	32	52.5
>3 times/week	3	4.9
Never	26	42. 6

### Physical activity

Based on IPAQ scores calculated with combined light-intensity PA (walking) and moderate-intensity or vigorous-intensity PA, 28/59 (47.5%) patients in our cohort reported low current PA, whereas 11 (18.6%) individuals were classified as highly active, according to the minimum WHO recommendation of 150–300 minutes/week of moderate-intensity aerobic PA or at least 75–150 minutes of vigorous-intensity PA or an equivalent combination of moderate- and vigorous-intensity PA throughout the week [24].

**Fig 1:**
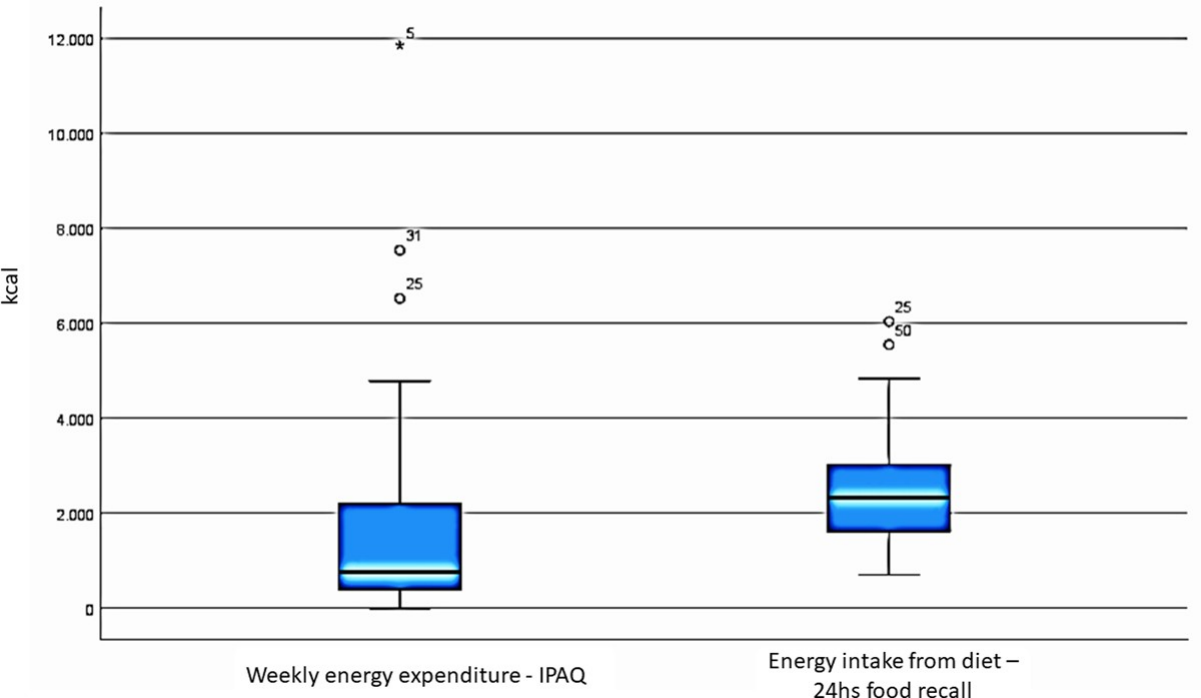
Physical activity expressed in weekly energy expenditure (A) and Energy intake from diet (B) in the study cohort.

The overall weekly energy expenditure was calculated transforming the metabolic equivalents (MET) assessed with IPAQ into kilocalories, using the Heymsfield formula [25] Despite a broad range of variation and existing outliers, the median weekly energy expenditure was estimated in about 1,000 kcal. Fig 1.

### Energy intake from diet

Assessing patients’ energy intake from diet is important to evaluate energy balance. Based on information retrieved from the 24-hour food recall, we calculated a mean intake of about 2,200 kcal, with individual values ranging between 600 to 4,600 kcal, after excluding outliers (6,000 kcal), as shown in Fig 1B.

### Social support for PA

Social support scores were calculated separately according to the type of activity (light-intensity PA (LPA)—walking, moderate-intensity (MPA) and vigorous-intensity (VPA) and the involved supporters (family members or people living in the patient’s household, and people living elsewhere) Table 2. Although an overall high level of social support for PA was found (45/58 patients, 77.6%), it should be highlighted that support of family members for walking (LPA) or for MPA or VPA was low: 79.3% reported never walking in their company, whereas 72.4% and 74.1%, respectively, were never invited or encouraged by them to walk. Likewise, 89.7% reported never performing MPA or VPA in their company, 81.0% and 79.3%, respectively, were never invited or encouraged by them to engage in MPA or VPA. Table 2

**Table 2:** Social support and self-efficacy scores for physical activity in the study cohort.

Variable	N	Standard deviation	Minimum	Median	Maximum
Social support	58	5.4	16	30	36
Self-efficacy					
walking	58	1.2	5	7	10
moderate to intense PA	58	16	5	7	10
total score	58	2.2	10	14	20

### Self-efficacy for PA

Self-efficacy for PA was described separately according to the type of activity (light-intensity PA—walking, and moderate to vigorous-intensity PA) and to the different contextual scenarios or domains (physical status, mood, availability of time, weather conditions). Based on the resulting scores (Table 2), self-efficacy for PA in our cohort was characterized as medium for 42 (72.4%) patients, whereas 14 (24.1%) and only 2 (3.5%), respectively, were classified as having high and low self-efficacy.

### Standardized physical evaluation

As for anthropometric measures, the median BMI (23.3 kg/m^2^) and waist-hip ratios of our patients fell within normal ranges. Table 3

**Table 3:** Anthropometric measures in the study cohort.

Variables	Median (IQR)
Body mass index (kg/m^2^)	23.32 (22.24–25.50)
Waist circumference (cm)	81.65 (75.50–85.31)
Abdomen circumference (cm)	84.50 (80.00–89.70)
Waist-hip ratio	0.84 (0.80–0.88)

IQR: Interquartile range.

### Medical charts

Medical chart review was possible for 60 patients and yielded infrequent references to PA in medical histories or recommendations. We could confirm that only 6 (10%) patients had PA addressed in their first consultation, and cumulatively it was registered in 27 (45%) charts along an 18-month clinical follow-up.

### Associations between PA, energy intake and psychosocial constructs (social support and self-efficacy for PA)

After adjusting for patients’ gender and age regression models revealed no associations between current PA and energy intake (p = 0.928), current PA and social support (p = 0.254) or between current PA and self-efficacy for PA (p = 0.466). Table 4

**Table 4:** Regression model results with Physical Activity (PA) as dependent variable and energy intake from diet, social support and self-efficacy for physical activity, gender, and age as explanatory variables.

Variables	Parameter estimate	Standard error	Test statistic	p
**PA/energy intake**				
Intercept	3051.9	946.8	3.22	0.001
Age	-41.3	21.5	-1.92	0.054
Gender (Female)	-458.5	554.6	-0.83	0.406
Self-efficacy for PA	0.01	0.2	0.09	0.928
**PA/Social support**				
Intercept	3487.5	1012.0	3.45	0.001
Age	-29.9	18.6	-1.61	0.107
Gender (Female)	-342.7	460.4	-0.74	0.460
Self-efficacy for PA	-34.9	30.5	-1.14	0.254
**PA/Self-efficacy**				
Intercept	2937.6	1081.2	2.72	0.007
Age	-33.6	18.1	-1.86	0.063
Gender (Female)	-419.5	459.4	-0.91	0.363
Self-efficacy for PA	-49.4	68.1	-0.73	0.466

However, we found an inverse association between energy intake and self-efficacy for PA, i.e., the higher the confidence the patient had in engaging in PA, the lower his/her energy intake was. Table 5

**Table 5:** Regression model results with energy intake as dependent variable and self-efficacy for Physical Activity (PA), gender, and age as explanatory variables.

Variable	Parameter estimate	Standard error	Test statistic	p
Intercept	3983.1	1078.1	3.69	<0.001
Age	6.8	16.5	0.41	0.682
Gender (Female)	-95.8	418.9	-0.23	0.818
Self-efficacy for PA	-127.1	62.1	-2.05	**0.040**

## Discussion

The increased survival of PLH under effective ART has brought about the need to reorganize HIV care to meet novel clinical demands. In this new scenario, a more comprehensive care approach is required to target health promotion and adoption of healthier lifestyles including PA. This task extends the scope of interventions from an exclusive medical perspective to incorporate a broader interdisciplinary view, involving other professionals, such as psychologists, dietitians, social workers, and physical education professionals.

In our study, we addressed the need of incorporating PA in the comprehensive care provided to PLH and the challenges for its effectiveness from the perspective of patients followed up at a university HIV outpatient clinic in the city of São Paulo, Brazil. Our option to evaluate this relevant issue among PLH starting ART without body changes and metabolic abnormalities was due to the fact the PA has already been well recognized as useful to treat these long-term adverse effects of chronic HIV infection and ART [7–13, 26]. In contrast, our main interest was to understand to what extent promotion of PA could benefit individuals at early stages of HIV infection and what services could do to maximize intervention uptake.

Our cohort was predominantly composed of adult males, with a significant share of non-white patients, in accordance the current epidemiological features of the Brazilian HIV epidemic [27]. As for their engagement in PA, our results underscored a low activity profile, below WHO recommendations of at least 150–300 minutes/week [24], with a median weekly energy expenditure of 1,000 kcal. A similar pattern of PA was reported among PLH in the city of Santarem, in the Brazilian Amazon region, with a weekly energy expenditure of 1,645 kcal [28]. Likewise, in a meta-analysis of 24 studies investigating PA rates among 3780 PLH, Vancampfort et al. [29] showed that only about 50% engaged in more than 150 minutes/week of moderate-intensity PA. Findings from these studies sharply contrast to those reported in an outpatient clinic in Bahia, Brazil, among patients not infected with HIV [30]. In that study when investigating PA as a protective factor against adult diabetes, an energy expenditure of 4,782 kcal/week was shown. Altogether, results from these studies reinforce the need to promote PA among PLH.

When contrasting the median energy intake (2,322 kcal) with energy expenditure in our cohort, we concluded they would lead to a significant energetic imbalance. It is therefore crucial to describe how potential facilitators of behavioral change can be used to help patients adopt healthier habits and, more specifically, increase their engagement in PA. For this evaluation, we assessed the validated psychosocial constructs of Social Support and Self-efficacy for PA. In this regard, although a significant proportion of our cohort exhibited high scores of social support for PA, support of family members for walking or for engaging in moderate to vigorous physical activity was low. Moreover, no association was found between social support scores and currently performed PA after controlling for patients’ gender and age. An important aspect to consider in this scenario was described among adults on ART in Ohio, USA [31], among whom the quality of social relations turned out more relevant than their density and quantity. Social belonging and social capital were shown significantly associated with higher adherence to ART and to the functional and self-satisfaction domains of patients’ quality of life in that cohort. In contrast, no association was demonstrated between patients’ social network and favorable health outcomes, neither between any variable related to social resources and engagement in PA. We can thus speculate that the quality of social interaction between PLH and their caregivers can be used to catalyze the effect of the social support they may have, for their own benefit.

Self-efficacy for PA is understood as the level of belief/confidence the individual has in performing an activity, and as such can be applied to predict how likely behavior change towards PA is [21]. In our cohort, self-efficacy was found medium, and not associated with reported PA after controlling for gender and age. It is important to understand why patients’ self-efficacy for PA did not effectively translate into activity. For this reflection, one should explore what prevents patients with other chronic conditions from adopting an active behavior. Physical barriers, such as fatigue and low physical energy were prominent in a broad study review involving patients with chronic renal disease from several countries [32]. Moreover, social aspects may also be important, as demonstrated among hemodialysis patients in São Paulo [33]. In that report, 37.8% identified lack of company as the leading barrier to exercise in leisure time. Finally, in a qualitative study based on semi-structured interviews of PLH, Gray et al. [34] defined three distinct categories of barriers: physical barriers that include symptoms such as fatigue, breathlessness, stomach problems, muscular and joint pain, and severe weight loss; those of psychological origin, including negative self-perception, perception of PA-associated risk and lack of motivation for PA; and social barriers, such as lack of social support, lack of time, financial constraints, unfavorable weather conditions and no offer of PA adapted to individual needs. In terms of social support, this last study suggested caregivers played conflicting roles, sometimes acting overprotectively with patients’ families to discourage engagement in PA or facilitating PA by reinforcing its safety and usefulness for PLH.

In a different perspective we evaluated the association between patients’ self-efficacy for PA and their calorie intake, calculated from the 24-hour food recall, and found an inverse association between these variables, after adjusting for gender and age, i.e, the higher the confidence patients had of engaging in PA, the lower their calorie intake. We hypothesize that patients may have planned to eat less calories as they were expecting to adopt healthier habits that included performing PA. However, for unknown reasons this latter intent did not translate into practice.

Health professionals engaged in HIV care should therefore intensify motivation strategies, reframing promotion of PA as an important component of comprehensive care. HIV caregivers are perceived as important actors of health promotion, who have frequent opportunities to integrate exercise into routine care of PLH [35, 36]. Nevertheless, results from our study show that such valuable opportunities are often being missed, as PA is not being properly addressed, even when followed-up at a reference HIV clinic. We believe members of the multi-professional team should be encouraged to effectively take their shared responsibility in this regard.

Limitations of our study should however be considered. Although 38 (62.3%) subjects were included from 2014 to 2016 and the remaining 23 (37.7%) from 2017 to 2018, inclusion criteria were kept the same throughout the study. Moreover, participants’ socio-demographic profile (gender, age, income, years of schooling), and reported smoking and alcohol use did not differ significantly between the two recruitment periods (S1 Table), rendering selection bias unlikely. However, the study single-center design and limited sample size preclude broad generalizations of results, given the particular features of our clinical setting and its clientele. Future multi-center studies are thus warranted to enable further conclusions regarding the incorporation of PA interventions for PLH starting ART and their implications for public policies.

Furthermore, we should bear in mind that the psychosocial constructs of social support and self-efficacy for PA may not have been able to consider the multiple and complex barriers and challenges PLH face regarding behavior change. Both constructs emerged from the Socio-Cognitive Theory (SCT) framework [37], in which the central focus is to evaluate the capacity of the individual in organizing and planning his/her actions to improve performance in the health promotion context; in our case, adopting healthier lifestyles that include PA. These constructs address relevant dimensions concerning behavior change, such as: the rational recognition by the individual of advantages and disadvantages of the current and the desired behavior, his/her self-perception before, during and after behavior change, the perception of others about the behavior to be changed, the individual’s self-confidence about coping with the difficulties in changing behavior with no need to resume the previous unhealthy behavior, and identifying and using social support for the desired change [38]. However, the SCT framework disregards intersubjective contexts and living conditions that may significantly affect health promotion and disease prevention, as highlighted by the proponents of the Vulnerability and Human Rights frameworks [39]. PLH are particularly recognized as living under intertwining and overlapping vulnerability contexts that include gender inequalities, social exclusion, stigma, and discrimination. In such a scenario, socio-cognitive psychosocial constructs may be limited as predictors of behavior change among PLH who are experiencing complex social dynamics that may hinder adoption of healthier lifestyles, and specifically, of PA.

Understanding care as the ultimate result of a fruitful interaction between patient and caregiver, in which the aims of interventions and the means how to use them are jointly defined using scientific and non-scientific knowledge and experiences of both patient and caregiver [39], we propose to adopt PA as a significant health promotion tool for PLH to be systematically incorporated in their routine care. As highlighted by these authors, the effectiveness of such interventions in the comprehensive care of these individuals will depend on how barriers are overcome, and resources mobilized to target healthy habits as part of the life projects of each individual and those of its peers and communities. In this comprehensive approach, being physically active would then successfully become part of one’s own life project in connection with others the individual feels he/she belongs to.

## Conclusions

Our results highlight a scenario in which Brazilian PLH starting antiretroviral report low engagement in PA and health professionals do not address PA in patient routine care as often as necessary. Despite exhibiting high-social support and medium self-efficacy for PA, these psychosocial constructs were not associated with current PA. Nevertheless, social support and self-efficacy scores still point out for the possibility of investing in behavioral change strategies aiming at increasing PA in this population. To maximize uptake of PA interventions and their impact healthcare professionals should therefore engage in dialogue with PLH to build customized care plans in which specific individual needs and vulnerabilities are taken into account along with current patterns of PA, calorie intake, health conditions, social support and self-efficacy for PA.

## Supporting information

S1 Table Cohort characteristics according to recruitment period. (DOCX)

## References

[1] Deeks SG, Lewin SR, Havlir DV. The end of AIDS: HIV infection as a chronic disease. Lancet. 2013;382(9903):1525–1533. doi: 10.1016/S0140-6736(13)61809-7 24152939 PMC4058441

[2] Chirch LM, Hasham M, Kuchel GA. HIV and aging: a clinical journey from Koch’s postulate to the chronic disease model and the contribution of geriatric syndromes. Curr Opin HIV AIDS. 2014;9(4):405–411. doi: 10.1097/COH.0000000000000074 24824883

[3] Risher KA, Kapoor S, Daramola AM, Paz-Bailey G, Skarbinski J, Doyle K, et al. Challenges in the evaluation of interventions to improve engagement along the HIV care continuum in the United States: a systematic review. AIDS Behav. 2017; 21(7):2101–2123. doi: 10.1007/s10461-017-1687-8 28120257 PMC5843766

[4] Fields-Gardner C, Fergusson P, American Dietetic Association, Dietitians of Canada. Position of the American Dietetic Association and Dietitians of Canada: nutrition intervention in the care of persons with human immunodeficiency virus infection. J Am Diet Assoc. 2004;104(9):1425–1441. doi: 10.1016/j.jada.2004.07.012 15354161

[5] Zonta MB, Almeida SM, Carvalho MTM, Werneck LC. Functional assessment of patients with AIDS disease. Braz J Infect Dis. 2003;7(5):301–306. doi: 10.1590/s1413-86702003000500004 14552739

[6] Erlandson KM, Schrack JA, Jankowski CM, Brown TT, Campbell TB. Functional impairment, disability, and frailty in adults aging with HIV-infection. Curr HIV/AIDS Rep. 2014;11(3):279–290. doi: 10.1007/s11904-014-0215-y 24966138 PMC4125474

[7] O’Brien KK, Tynan A-M, Nixon SA, Glazier RH. Effectiveness of progressive resistive exercise (PRE) in the context of HIV: systematic review and meta-analysis using the Cochrane collaboration protocol. BMC Infectious Diseases. 2017;17(1):268. doi: 10.1186/s12879-017-2342-8 28403830 PMC5389006

[8] O’Brien KK, Tynan A-M, Nixon SA, Glazier RH. Effectiveness of aerobic exercise for adults living with HIV: systematic review and meta-analysis using the Cochrane Collaboration protocol. BMC Infectious Diseases. 2016;16:182. doi: 10.1186/s12879-016-1478-2 27112335 PMC4845358

[9] Brito-Neto JG, Andrade MF, Almeida VD, Paiva DCC, Morais NM, Bezerra CM, et al. Strength training improves body composition, muscle strength and increases CD4+ T lymphocyte levels in people living with HIV. Infect Dis Rep. 2019;11:7925. doi: 10.4081/idr.2019.7925 31205641 PMC6547026

[10] Perry AC, La Perriere A, Klimas N. Acquired immune deficiency syndrome (AIDS). In: Durstine JL, Moore GE, editors. ACSM’s exercise management for persons with chronic diseases and disabilities. 2^nd^ ed. Champaign, IL: Human Kinetics; 2003.

[11] Terry L, Sprinz E, Stein R, Medeiros NB, Oliveira J, Ribeiro JP. Exercise training in HIV-1-infected individuals with dyslipidemia and lipodystrophy. Med Sci Sports Exerc. 2006;38(3):411–417. doi: 10.1249/01.mss.0000191347.73848.80 16540826

[12] Haskell WL, Lee IM, Pate RR, Powell KE, Blair SN, Franklin BA et al. Physical activity and public health: updated recommendation for adults from the American College of Sports Medicine and the American Heart Association. Circulation. 2007; 116(9):1081–1093. doi: 10.1161/CIRCULATIONAHA.107.185649 17671237

[13] Gomes-Neto M, Saquetto MB, Alves IG, Martinez BP, Vieira JPB, Brites C. Effects of exercise interventions on aerobic capacity and health-related quality of life in people living with HIV/AIDS. Physical therapy. 2021;Mar 10. 10.1093/ptj/pzab092. 33704496

[14] Brasil. Ministério da Saúde. Recomendações para a prática de atividades físicas para pessoas vivendo com HIV e aids. Brasília (DF): Ministério da Saúde, Secretaria de Vigilância em Saúde, Departamento de DST, Aids e Hepatites Virais; 2012. Available from: http://www.aids.gov.br/pt-br/pub/2012/recomendacoes-para-pratica-de-atividades-fisicas-para-pessoas-vivendo-com-hiv-e-aids-2012. Accessed June 7, 2021.

[15] Brasil. Ministério da Saúde. Cuidado integral a pessoas que vivem com HIV pela atenção básica. Brasília (DF): Ministério da Saúde, Secretaria de Vigilância em Saúde, Departamento de DST, Aids e Hepatites Virais; 2016. Available from: http://www.aids.gov.br/pt-br/pub/2016/cuidado-integral-pessoas-que-vivem-com-hiv-pela-atencao-basica. Accessed June 7, 2021.

[16] Brasil. Ministério da Saúde. Secretaria de Vigilância em Saúde. Departamento de Análise em Saúde e Vigilância de Doenças Não Transmissíveis. Vigitel Brasil 2019: vigilância de fatores de risco e proteção para doenças crônicas por inquérito telefônico: estimativas sobre frequência e distribuição sociodemográfica de fatores de risco e proteção para doenças crônicas nas capitais dos 26 estados brasileiros e no Distrito Federal em 2019. Brasília (DF): 2020. 137p. Available from: https://www.gov.br/saude/pt-br/centrais-de-conteudo/vigitel-brasil-2019-vigilancia-fatores-risco-pdf/view. Accessed June 7, 2021.

[17] Santos ECM, Florindo AA, Santos AV, Picone CM, Dias TG, Segurado AC, Multicomponent physical activity program to prevent body changes and meatbolic disturbances associated with antiretroviral therapy and improve quality of live of people living with HIV. Clinics. 2021;76:e2457. doi: 10.6061/clinics/2021/e2457 33787675 PMC7955151

[18] Matsudo S, Araújo T, Matsudo V, Andrade D, Andrade E, Oliveira LC, et al. Questionário internacional de atividade física (IPAQ): estudo de validade e reprodutibilidade no Brasil / International physical activity questionnaire (IPAQ): study of validity and reability in Brazil. Rev Bras Ativ Fis Saúde. 2001;6(2):5–18.

[19] Gibson RS. Principles of nutritional assessment. 2^nd^ ed. New York: Oxford University Press; 1990.

[20] Reis MS, Reis RS, Hallal PC. [Validity and reliability of a physical activity social support assessment scale]. Rev Saúde Pública. 2011;45(2):294–301. doi: 10.1590/s0034-89102011000200008 21412569

[21] Rech CR, Sarabia TT, Fermino RC, Hallal PC, Reis RS. [Psychometric properties of a self-efficacy scale for physical activity in Brazilian adults]. Rev Panam Salud Publica. 2011;29(4):259–266. doi: 10.1590/s1020-49892011000400007 21603771

[22] World Health Organization (WHO). Obesity: preventing and managing the global epidemic. Report of a WHO consultation. World Health Organ Tech Rep Ser. 2000;894:i-xii,1–253. 11234459

[23] World Health Organization (WHO). WHOQOL User Manual. Programme on Mental Health. 2012. Available from: https://www.who.int/healthinfo/survey/whoqol-qualityoflife/en/. Accessed June 7, 2021.

[24] World Health Organization (WHO). WHO guidelines on physical activity and sedentary behaviour. Available from: https://www.who.int/publications/i/item/9789240015128. Accessed June 7, 2021. 33369898

[25] Heymsfield S, Lohman TG, Wang ZM, Going SB. Human body composition: Methods and findings. 2nd ed. Champaign, IL: Human Kinetics; 2005.

[26] Ozemek C, Erlandson KM, Jankowski CM. Progress in Cardiovascular Diseases. 2020;63:178–183. doi: 10.1016/j.pcad.2020.01.005 32014512

[27] Brasil. Ministério da Saúde. Boletim epidemiológico HIV/aids. Secretaria de Vigilância em Saúde. Brasília (DF): Departamento de DST, Aids e Hepatites Virais; 2019. Available from: http://www.aids.gov.br/pt-br/pub/2019/boletim-epidemiologico-de-hivaids-2019. Accessed JUne 7, 2021.

[28] Gouvea-e-Silva LF, Said RC, Kietzer KS, Freitas JJS, Xavier MB. Nível de atividade física e síndrome lipodistrófica em pacientes com HIV/aids. Rev Bras Med Esporte. 2016;22(2):147–152.

[29] Vancampfort D, Mugisha J, De Hert M, Probst M, Firth J, Gorczynski P, et al. Global physical activity levels among people living with HIV: a systematic review and meta-analysis. Disabil Rehabil. 2018;40(4):388–397. doi: 10.1080/09638288.2016.1260645 27929355

[30] Almeida LAB, Pitanga FJG, Freitas MM, Pitanga CPS, Dantas EHM, Beck CC. [Caloric expenditure of different domains of physical activity as predictors of the absence of diabetes in adults]. Rev Brasil Med Esporte. 2012; 18(1):17–21.

[31] Webel AR, Sattar A, Schreiner N, Phillips JC. Social resources, health promotion behavior, and quality of life in adults living with HIV. Appl Nurs Res. 2016;30:204–209. doi: 10.1016/j.apnr.2015.08.001 27091279 PMC4838775

[32] Hannan M, Bronas UG. Barriers to exercise for patients with renal disease: an integrative review. J Nephrol. 2017;30(6):729–741. doi: 10.1007/s40620-017-0420-z 28689231 PMC8171436

[33] Rosa CSC, Bueno DR, Souza GD, Gobbo LA, Freitas IF Jr, Sakkas GK, et al. Factors associated with leisure-time physical activity among patients undergoing hemodialysis. BMC Neph. 205;16:192. doi: 10.1186/s12882-015-0183-5 26613791 PMC4662813

[34] Gray L, Schuft L, Bergamaschi A, Filleul V, Colson SS, d’Arripe-Longueville F. Perceived barriers to and facilitators of physical activity in people living with HIV: A qualitative study in a French sample. Chronic Illn. 2019;Feb26:1742395319826638. doi: 10.1177/1742395319826638 30808204

[35] Montoya JL, Jankowski CM, O’Brien KK, Webel AR, Oursler KK, Henry BL, et al. Evidence-informed practical recommendations for increasing physical activity among people living with HIV. AIDS 2019;33(6):931–939. doi: 10.1097/QAD.0000000000002137 30946147 PMC6457127

[36] Webel AR, Perazzo JD, Dawson-Rose C, Smith C, Nicholas PK, Rivéro-Mendez M. A multinational investigation on the pespectives and drivers of exercise and dietary behaviors in people living with HIV. Appl Nursing Res. 2017;37:13–18. 10.1016/j.apnr.2017.07.002 PMC5669066 28985914

[37] Janevic MR, Connell CM. Individual theories. In: Hilliard ME, Rieckert KA, Ockene JK, Pbert L. The Handbook of Health Behavior Change. New York: Springer Publishing Company, 2018; pp. 3–24.

[38] Mendonça LGT. Modelos teóricos como subsídios da prática da promoção da saúde em DST/aids no quadro da vulnerabilidade e dos direitos humanos. In: Paiva V, Calazans G, Segurado A, eds. Vulnerabilidade e direitos humanos–prevenção e promoção da saúde: entre Indivíduos e comunidade–Livro II, Curitiba: Juruá, 2012; pp. 73–100.

[39] Ayres JRCM, Paiva V, França Jr. I. Conceitos e práticas de prevenção: da história natural da doença ao quadro de vulnerabilidade e direitos humanos. In: Paiva V, Ayres JRCM, Buchalla C, eds. Vulnerabilidade e direitos humanos—prevenção e promoção da saúde: da doença à cidadania. Livro I, Curitiba: Juruá, 2012; pp. 71–94.

