# Supplementary material for: Incorporating physical activity in the comprehensive care of people living with HIV starting antiretroviral therapy: Insights from a specialized care setting in São Paulo, Brazil
Source: PLoS One. 2021 Jul 1;16(7):e0254168. doi: 10.1371/journal.pone.0254168 (PMC8248735; doi:10.1371/journal.pone.0254168)
Supplement: S1 Table — (DOCX) [file pone.0254168.s001.docx]

**S1 Table. Cohort characteristics according to recruitment period.**

| **Variables** | **Recruitment 1**  **(2014-5)**  **N = 38 (62.3%)** | **Recruitment 2**  **(2017-8)**  **N = 23 (37.7%)** | **p** |
| --- | --- | --- | --- |
| Gender |  |  |  |
| male | 33 (86.8) | 20 (87.0) | 0.654^a^ |
| female | 5 (13.2) | 3 (13.0) |  |
| Age – mean (SD) | 32.7 (9.1) | 33.6 (7.3) | 0.681^b^ |
| Income [reais] – median (IQR)* | 1950 (1050-3600) | 2100 (1600-3000) | 0.713^d^ |
| Schooling [years] – median (IQR)** | 12 (11-15) | 13 (11-15) | 0.477^d^ |
| Smoking |  |  | 0.698^c^ |
| yes | 13 (34.2) | 9 (39.1) |  |
| no | 25 (65.8) | 14 (60.9) |  |
| Alcohol use |  |  |  |
| yes | 23 (60.5) | 12 (52.2) | 0.521^c^ |
| no | 15 (39.5) | 11 (47.8) |  |

Categorical variables are presented in absolute numbers (%), whereas numerical variables are presented in mean (SD) or median (IQR) values, as appropriate.

*One missing value for recruitment 1 and one for recruitment 2.

**One missing value for recruitment 1.

^a^Fisher exact test; ^b^t-Test; ^c^chi-square test; ^d^Mann-Whitney test.
